# Supplementary material for: Functional Characterisation of the Transcription Factor GsWRKY23 Gene from Glycine soja in Overexpressed Soybean Composite Plants and Arabidopsis under Salt Stress
Source: Plants (Basel). 2023 Aug 23;12(17):3030. doi: 10.3390/plants12173030 (PMC10490167; doi:10.3390/plants12173030)
Supplement: Supplementary file 1 [file plants-12-03030-s001.zip › plants-2577972-supplementary.pdf]

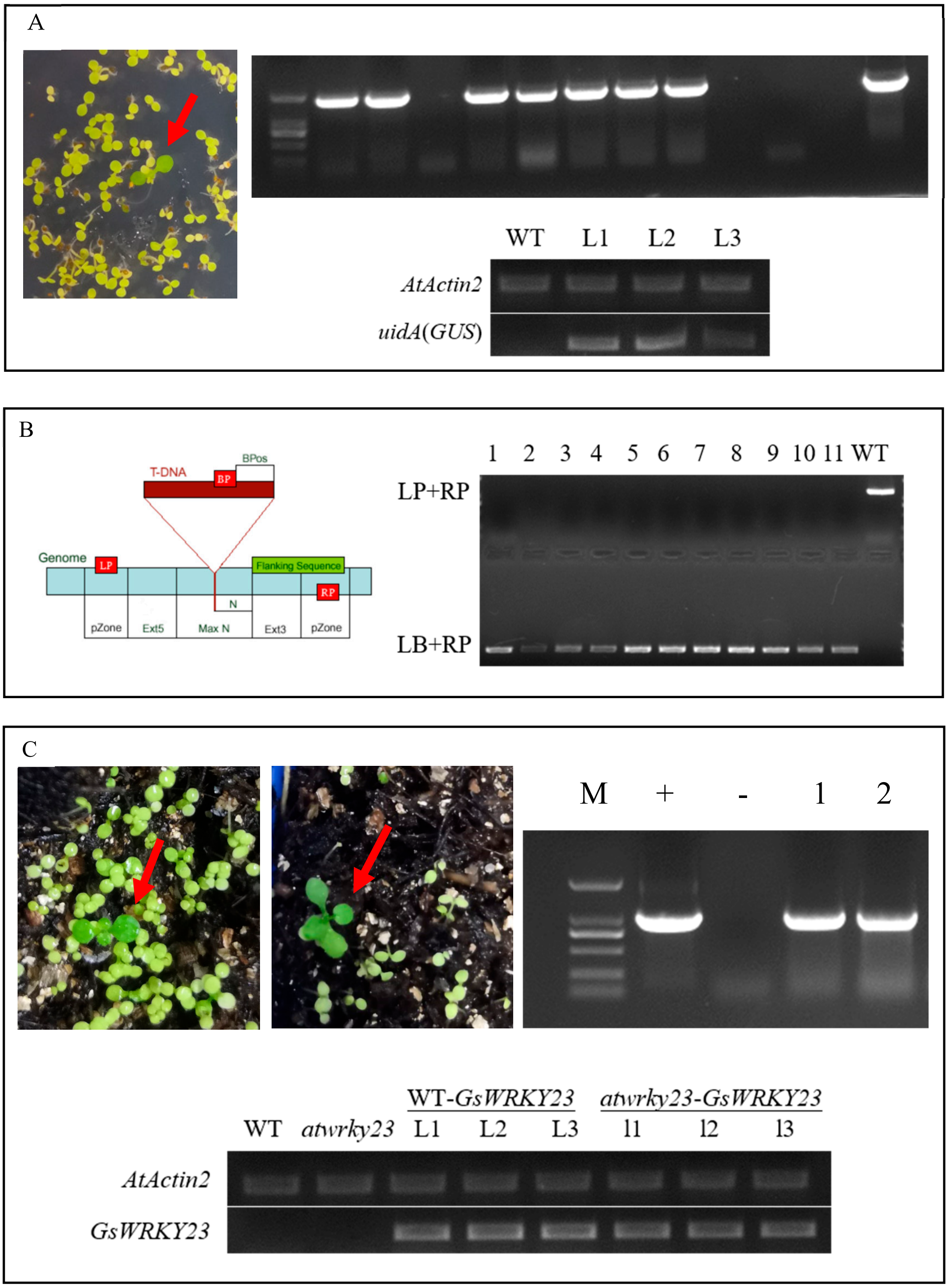

Figure S1 (A) Screening and PCR identification of *GsWRKY23pro*::GUS-transgenic *Arabidopsis* seedlings using 25 mg/L kanamycin and PCR identification. (B) Identification of *Arabidopsis atwrky23* mutant. (C) Screening and PCR identification of *GsWRKY23*-transgenic *Arabidopsis* WT and *atwrky23* seedlings. The red arrows indicate the resistant seedlings.

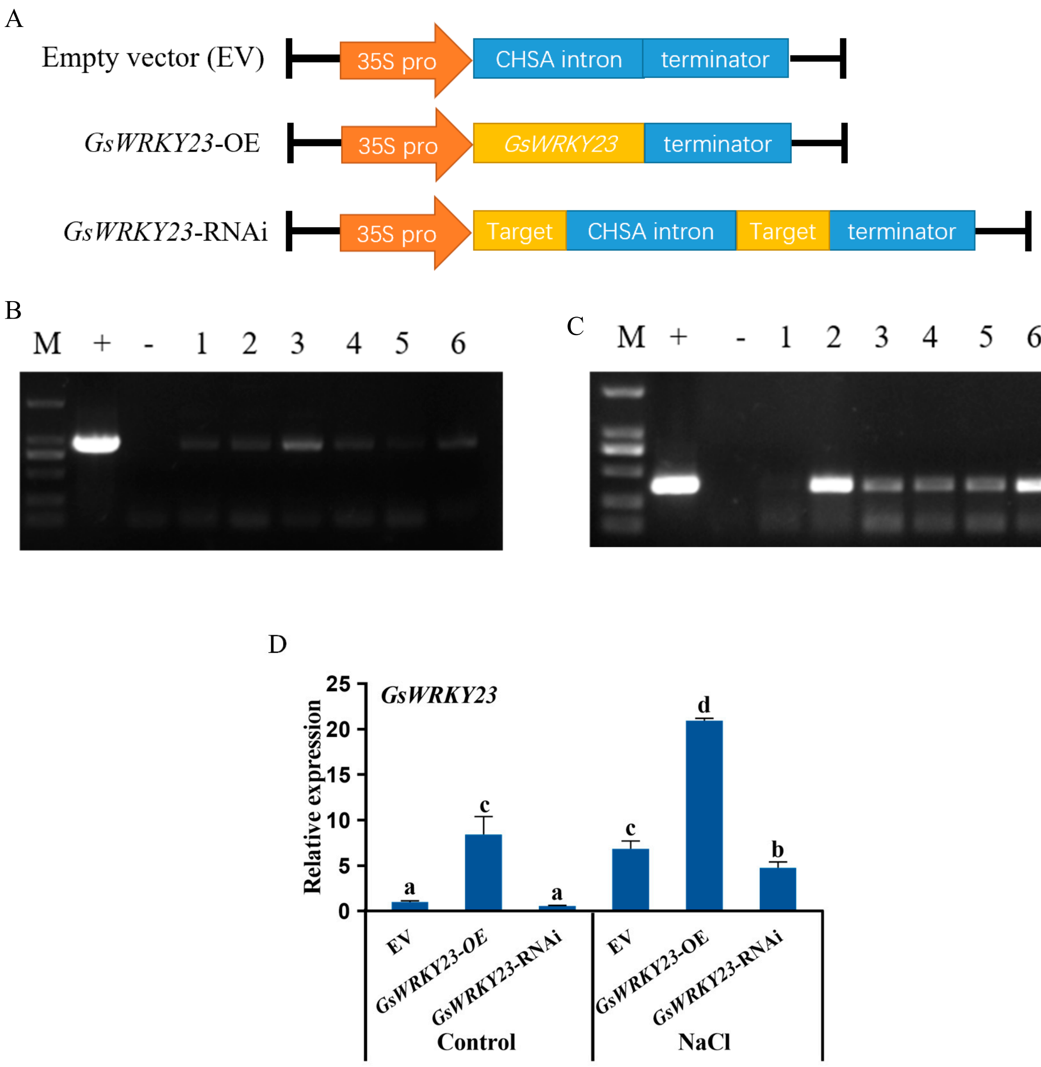

Figure S2 (A) Construction diagram of Gs*WRKY23*-OE and -RNAi vectors. (B) PCR identification of positive seedlings of *GsWRKY23*-OE soybean hairy root-composite seedlings. (C) PCR identification of positive seedlings of *GsWRKY23*-RNAi soybean hairy root-composite seedlings. (D) Detection of *GsWRKY23* gene expression levels in the roots of *GsWRKY23*-OE and *GsWRKY23*-RNAi hairy-root composite soybean plants under control and 120 mM NaCl solutions for 6 h.

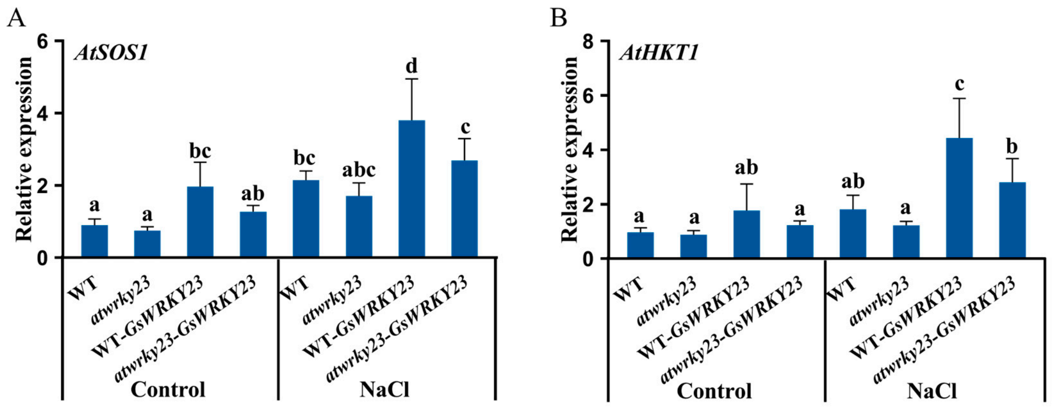

Figure S3 Expression levels of *AtSOS1* and *AtHKT1* in the shoots of *GsWRKY23*-transgenic *Arabidopsis* WT and *atwrky23* seedlings under 120 mM NaCl solutions for 6 h. (A) *AtSOS1*, (B) *AtHKT1*. Means ± SD are shown (n = 3), different letters indicate groups with statistically significant differences using Duncan's test ($P \leq 0.05$).

Table S1 Primers used in this study

Prime names	Sequences (5'→3')	Purposes
q*GsUBI3*-F	GTGTAATGTTGGATGTGTTCCC	Internal reference in *Glycine soja*.
q*GsUBI3*-R	ACACAATTGAGTTCAACACAAACCG	
q*GsWRKY23*-F	CGTGCAACCTCATCACTCTAC	qRT-PCR analysis
q*GsWRKY23*-R	ATCACTGGACGCGGATGAAA	
q*GsHKT1-1*-F	TTTCACTTCCATGCTCGACCT	
q*GsHKT1-1*-R	TGATTTGCATGATTTGTGGGTGG	
q*GsNHX1*-F	CCTCCGTGGTCTCCATGAAC	
q*GsNHX1*-R	TTTCACCTGAAACCCGGCAT	
q*GsSOS1*-F	GGTACTCATCATCGGCTGGG	
q*GsSOS1*-R	ACCAGGGCCAGCTAGTAAGA	
q*GsNRT2*-F	AGCTCTTGCTACGTACTTGAA	
q*GsNRT2*-R	TTGGCTGTGGTGTCTGTAGG	
q*GsCLC1*-F	TGTGGGAGGAGTTCTCTTTGC	
q*GsCLC1*-R	AGAGGCCCTTAGCACTACCA	
q*GsCLC-c2*-F	CGCGATGTTGTTTCAGGTCC	
q*GsCLC-c2*-R	AGCCCACATAACTCTGGTGC	
q*GsCLC-b2*-F	TGGGAAATGCGGCCTTTTTG	
q*GsCLC-b2*-R	AAAGGCTTCCCAATACCCCG	
q*AtHKT1*-F	CCTCTACGTCTCCTATTTCACC	
q*AtHKT1*-R	ACTAAGAACCACCGAGTACAAG	
q*AtSOS1*-F	ATTTTGATGCAGTCAGTGGATG	
q*AtSOS1*-R	GCAAGCAGATTCTAGTCTTTCG	
GsWRKY23pro-F	AACTATGTCAGCATGCTTCTC	Cloning the promoter region of *GsWRKY23*
GsWRKY23pro-R	ATACCTAACAAGTTGATCTCTTAA	
GsWRKY23-F	ATGGAGAAGAAGGAGATGGCT	Cloning the CDS of *GsWRKY23*
GsWRKY23-R	CTACTCTTCTTTCAACATGTGTGAAG	
GFP-*GsWRKY23*-F	ATGGAGAAGAAGGAGATGGCT	Cloning the CDS of *GsWRKY23* without a stop codon
GFP-*GsWRKY23*-R	CTCTTCTTTCAACATGTGTGAAG	
GsWRKY23-RNAi-up-F	TTACTGGGTGTGCAGGACTATAG	Upstream target sequence of *GsWRKY23* for RNAi
GsWRKY23-RNAi-up-R	CTTATTCTGTTCATCATTGACTGC	
GsWRKY23-RNAi-down-F	CTTATTCTGTTCATCATTGACTGC	Downstream target sequence of *GsWRKY23* for RNAi
GsWRKY23-RNAi-down-R	TTACTGGGTGTGCAGGACTATAG	
AtActin2-F	AGGTATCGCTGACCGTATGA	Internal reference in *Arabidopsis*
AtActin2-R	GCTGAGGGAAGCAAGAATG	
mutant-*atwrky23*-LP	TGATCCGAACACAAAGACTCC	Identification of *Arabidopsis atwrky23* mutant
mutant-*atwrky23*-RP	TCCATTAAATTCGGCCTTTTC	
mutant-LB	ATTTTGCCGATTTCGGAAC	
